# Radiation Doses in Routine CT Examinations for Adult Patients in Saudi Arabia: A Systematic Review

**DOI:** 10.7759/cureus.64646

**Published:** 2024-07-16

**Authors:** Khaled Alenazi

**Affiliations:** 1 Radiological Sciences Department, College of Applied Medical Sciences, King Saud University, Riyadh, SAU

**Keywords:** volumetric ct dose index, adult, dose length product, diagnostic reference levels, computed tomography

## Abstract

Computed tomography (CT) is an important imaging technique that produces detailed cross-sectional images for diagnosing medical conditions. However, the associated radiation exposure raises concerns. Establishing diagnostic reference levels (DRLs) helps identify unusual radiation doses and optimize exposure while maintaining diagnostic image quality. The purpose of this systematic review is to review the radiation doses received by adult patients in the head, chest, abdomen, pelvis, abdomen-pelvis (AP), and combined chest, abdomen, and pelvis (CAP) CT scans in Saudi Arabia. A search was conducted in several databases including PubMed and Google Scholar to identify studies that have established DRLs or determined radiation dose for adult CT examinations. Only studies that specifically assessed DRLs in actual adult patients were considered for inclusion. Out of a total of 31 articles that were identified as eligible, 13 were included after a thorough screening process. The values of CTDI_v_, DLP, and effective doses were determined. The review discovered that CTDI_v_ and DLP were the most frequently used dosimetric quantities. The mean values in terms of CTDI_v_ for head, chest, abdomen, pelvis, AP, and CAP ranged from 40.67 to 61.80 mGy, 5.80 to 14.90 mGy, 8.60 to 16.15 mGy, 10.80 to 17.35 mGy, 14.10 to 16.84 mGy, and 12.00 to 22.94 mGy, respectively. The mean values in terms of DLP for head, chest, abdomen, pelvis, AP, and CAP ranged from 757 to 1212 mGy.cm, 243 to 657 mGy.cm, 369.5 to 549 mGy.cm, 379.6 to 593 mGy.cm, 658 to 940.43 mGy.cm, and 740 to 1493.8 mGy.cm, respectively. There is a fluctuation in radiation dose among CT centers, highlighting a need to provide proper education and training to radiographers. It is recommended to establish a universally accepted standardized protocol based on weight, equivalent diameter, or cross-sectional area for accurate comparisons with national and international DRLs.

## Introduction and background

Computed tomography (CT) scans are considered one of the most crucial radiological modalities for disease detection, using cross-sectional images [1,2]. The increasing use of CT in medicine has led to a rise in radiation exposure for patients and the general population, making it a significant factor in the overall radiation dose received [3,4]. CT scans have been a significant source of radiation exposure and contributed to a significant portion of the overall radiation dose from medical procedures [1]. For example, in Germany and the UK, CT scans account for around 35% and 47% of the collective dose, respectively [5,6].

The convenience and speed of imaging procedures in CT can sometimes lead to inappropriate use, where a large portion of body scanning is performed instead of focusing on a specific area of the body [7,8]. Despite the advancements in technology to lower patient doses during CT scans, the desire to obtain high-quality images that cover a larger portion of the patient's anatomy can have the opposite effect [8]. It has been observed that patients are being exposed to higher doses of radiation than required, and the image quality produced by CT scans often surpasses the level necessary for accurate diagnosis [9]. Therefore, there has been a strong focus on producing protocols and guidelines to minimize radiation dose used in CT scans to the least possible level [10].

The International Commission on Radiation Protection (ICRP) established three basic principles of radiation protection, which are justification, optimization, and dose limitation, to ensure that the potential risks associated with radiation exposure during CT imaging do not outweigh the benefits derived from CT procedure [11]. The dose length product (DLP) and the volumetric CT index are the fundamental radiation quantities in the CT control. The guidelines for establishing diagnostic reference levels (DRLs) recommend using the volumetric CT dose index (CTDI_v_) for a single slice and the DLP for the entire coverage volume as the dosimetric quantities [12-14].

The ICRP promotes the use of a DRL that represents a specific medical practice within a particular geographical region. The primary objective of establishing DRLs is to minimize radiation doses and enhance the image quality provided by medical practitioners [10,15-18]. The aim of this study is to conduct a systematic review of the published literature regarding patient radiation doses in routine CT examinations, compare the findings with DRLs, and assess whether patient doses have decreased or increased in more recent studies.

CT dosimetry involves using cylindrical phantoms containing four peripheral holes and one central hole for the placement of dosimeters [19]. The CTDI is measured with 100 mm-long pencil-like ionization chambers inserted into the phantom cavities. The weighted CTDI (CTDl_w_) is calculated using two measured CTDI values: one from the central cavity of the acrylic phantom and the other from the average of measurements at peripheral cavity positions (3, 6, 9, and 12 o'clock positions), as shown in the following formula [20,21]:

$$CTDI{w}(mGy)= \frac{1}{3}CTDI{center} + \frac{2}{3}CTDI{periphery}$$

The CTDI_v_ indicator is often used to address the impact of helical and axial doses when the slice spacing (I) deviates from the slice thickness (n x T), where n represents the number of slices and T represents the slice thickness [20]:

$$CTDI{v}(mGy)= CTDI{w} \left ( \frac{n x T}{I} \right )$$

To determine the total radiation deposited in the patient and assess radiation risk, the DLP is used as a product of CTDI_v_ and irradiated scan length (L) (cm) [21]:

$$DLP(mGy.cm)= CTDI{v} * L$$

The routine use of the DLP as a risk indicator is limited because it does not consider the radiosensitivity of organs within the irradiated tissues [1]. Thus, the ICRP introduced the concept of effective dose (ED), which takes into account the radiosensitivity of different organs within the body [1,11]. A reasonable estimate of the effective dose can be calculated using the following equation [9,21]:

$$ED(mSv)= DLP * CF$$

where CF is the conversion factor (mSv mGy^-1^ cm^-1^).

## Review

Methodology

**Figure 1:**
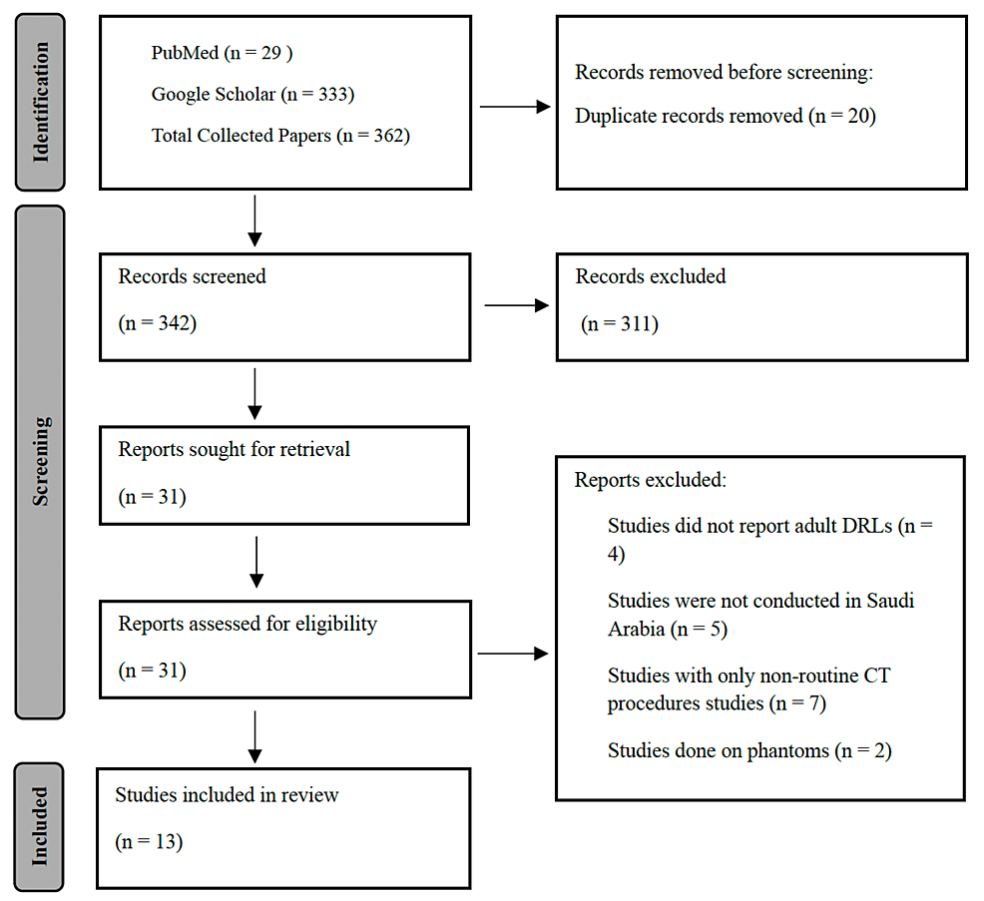
Preferred Reporting Items for Systematic Reviews and Meta-Analyses (PRISMA) flow chart showing how the articles were identified.

The review search was conducted on PubMed and Google Scholar databases, covering the period from 2014 to 2024. The necessary terms used were "diagnostic reference level in Saudi Arabia" along with "computed tomography." Further papers were identified by reviewing the references of the retrieved articles. The search strategy used the Preferred Reporting Items for Systematic Reviews and Meta-Analyses (PRISMA) flow chart in Figure 1. This systematic review concentrated on studies involving adult patients, and the dosimetric data were extracted for routine CT scans, including the head, chest, abdomen, pelvis, abdomen-pelvis (AP), and combined chest, abdomen, and pelvis (CAP) shown in Table 1. The exclusion criteria were articles focused on CT doses for pediatric examinations or phantom studies. The dosimetric quantities assessed in the studies included CTDI_v_, DLP, effective dose, and CTDI_w_ listed in Table 2. The mean and the range values were determined for each examination and compared to NDRLs proposed by the Saudi Food and Drug Authority (SFDA). The variations in dosimetric quantities among studies were assessed with respect to the year of publication.

**Table 1:** CT scans and frequency of the articles' reported patient doses.

CT exam	Number of studies
Head	5
Chest	8
Abdomen	4
Pelvis	4
Abdominopelvic	4
CAP	5

**Table 2:** Frequency of articles reporting the listed dosimetric quantities. CTDI_v _: volumetric CT dose index, DLP: dose length product

Quantity	Number of studies
CTDI_v_	12
DLP	12
Effective dose	9

Results

Thirty-one articles were eligible and were identified through the review search, and 18 articles were removed as they did not meet the review inclusion criteria. According to the data retrieved, the chest CT scan was the most frequently analyzed body part, as shown in Table 1. The reviewed studies utilized different dosimetric quantities, with DLP and CTDI_v_ being the most commonly employed. The reported effective dose was calculated using the following methodologies: 1) multiplying the DLP by the appropriate conversion factors and 2) inputting scan parameters into Monte Carlo applications, such as CT-Expo and ImPACT dose calculators. 

The studies selected in this review are summarized in Table 3. The average, range, second and third quartiles of CTDI_v_, DLP, and ED in the selected studies were given for each routine examination in Table 4. Figures 2-13 show the reported values of CTDI_v_ and DLP from each selected study sorted by CT examination.

**Table 3:** Summary of the selected studies AP: abdomen and pelvis, CAP: chest, abdomen, and pelvis

Reference	No. of health centers	No. of patients	Body part
Qurashi et al., 2015 [22]	24	550	Chest, AP, and CAP
Manssor et al., 2015 [23]	-	51	CAP
Sulieman et al., 2018 [24]	2	60	Head and chest
Alkhorayef, 2018 [25]	3	35	CAP
Taha et al., 2020 [26]	1	481	Head, chest, and CAP
Osman et al., 2021 [27]	2	313	Pelvis
Saeed et al., 2021 [28]	5	360	Chest, abdomen, and pelvis
Al-Othman et al., 2022 [29]	1	3000	Head, chest, and AP
Alashban et al., 2022 [30]	3	399	Head, chest, abdomen, pelvis, AP, and CAP
Alrehily et al., 2023 [31]	1	150	Head and chest
Almujally et al., 2023 [32]	1	1444	AP
Osman at el., 2023 [33]	2	313	Abdomen and pelvis
Ahmedet et al., 2024 [34]	2	428	Chest and abdomen

**Table 4:** Dosimetric quantities for various national studies *The mean values of the CTDIv and DLP were calculated from the mean values of health centers studied in this article. **The author referred to the DLP as the air kerma length product (PKL, CT). ^a^ Before applying the NDRL (with default parameters). ^b ^After applying NDRL (i.e., post-optimization with optimized parameters). AP: abdomen and pelvis, CAP: chest, abdomen, and pelvis, CTDI_v_: volumetric dose index, DLP: dose length product

Author	CT exam	CTDI_v_ (mGy)		DLP (mGy cm)			ED (mSv)		
		Mean (min-max)	3rd quartile	Mean (min-max)	2nd quartile	3rd quartile	Mean (min-max)	2nd quartile	3rd quartile
Qurashi et al., 2015 [22]	Chest	14.3 (3.2-31.2)	18	520 (101-1635)	-	630	-	-	-
	AP	14.1 (6.3-41.9)	15	685 (180-1772)	-	800	-	-	-
	CAP	15 (8.8-39.7)	16	1000 (299-2584)	-	1040	-	-	-
Manssor et al., 2015 [23]	CAP	22.94 (9.4-39.4)	-	1493.8 (587-2508)	-	-	21.2 (8.3-35.6)	-	-
Sulieman et al., 2018 [24]*	Head	50.47 (9.8-68.1)	-	921.17 (264.4-2107.4)	-	-	1.9 (0.6-4.4)	-	-
	Chest	11.65 (4.7-22.1)	-	529.1 (137.7-2483.1)	-	-	7.4 (0.5-34.8)	-	-
Alkhorayef, 2018 [25]**	CAP	12 (8.1-17)	-	740 (400.7-1100)	-	-	11.8 (6.4-17.1)	-	-
Taha et al., 2020 [26]	Head	61.8	-	1212	-	-	2.55	-	-
	Chest	13.1	-	657	-	-	9.21	-	-
	CAP	13.9	-	783	-	-	11.73	-	-
Osman et al., 2021 [27]	Pelvis	-	-	593	-	-	8.9	-	-
Saeed et al., 2021 [28]	Chest	14.48 (7.71-27.36)	-	-	-	-	-	-	-
	Abdomen	16.15 (10.84-17.27)	-	-	-	-	-	-	-
	Pelvis	17.35 (5.63-23.79)	-	-	-	-	-	-	-
Al-Othman et al., 2022 [29]	Head^a^	40.67	-	757	-	-	1.74	-	-
	Chest^a^	14.9	-	547	-	-	7.27	-	-
	AP^a^	16.84	-	658	-	-	10.2	-	-
	Head^b^	45.61		788			1.83		
	Chest^b^	10.40		393			4.19		
	AP^b^	12.20		583			8.72		
Alashban et al., 2022 [30]	Head	(5.72-5109)	-	(98.2-1163)	-	893.1	-	-	-
	Chest	(3.74-22.61)	-	(136.5-1124.13)	-	904.99	-	-	-
	Abdomen	(2.52-51.4)	-	(137.7-1403.66)	-	899.88	-	-	-
	Pelvis	(9.53-38.63)	-	(244.14-1205.3)	-	884.52	-	-	-
	AP	(2.54-17.16)	-	(6.43-893.6)	-	506	-	-	-
	CAP	(3.92-29.14)	-	(284.1-2216.7)	-	1199.67	-	-	-
Alrehily et al., 2023 [31]	Head	-	63	-	-	1187	-	-	-
	Chest	-	6	-	-	243	-	-	-
Almujally et al., 2023 [32]	AP	15 (15.02-15.9)	-	900 (701.52-1296.17)	-	-	14.18 (10.15-19.44)	-	-
Osman et al., 2023 [33]	Abdomen	11.5	-	369.5	-	407.5	5.6	-	-
	Pelvis	10.8	-	379.6	-	402.5	7.2	-	-
Ahmed et al., 2024 [34]	Chest	5.8 (3.4-9.5)	6.9	243 (189-410)	-	375	-	5.1	-
	Abdomen	8.6 (4.7-11.8)	7.8	549 (465-818)	-	747	-	21.1	-

**Figure 2:**
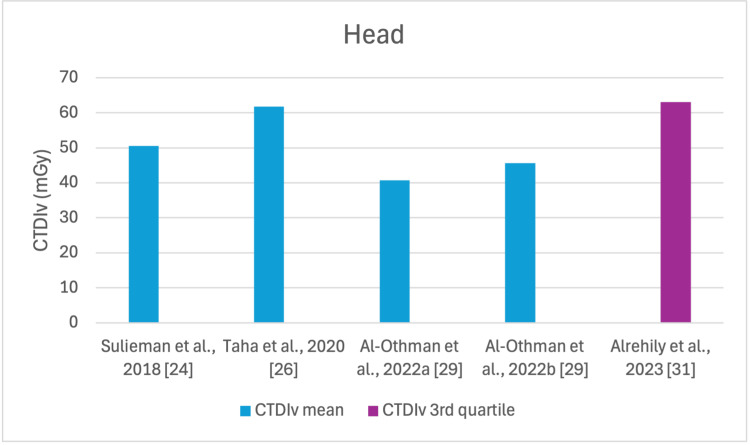
CTDIv reported by each study for the head examination CTDI_v_: volumetric dose index

**Figure 3:**
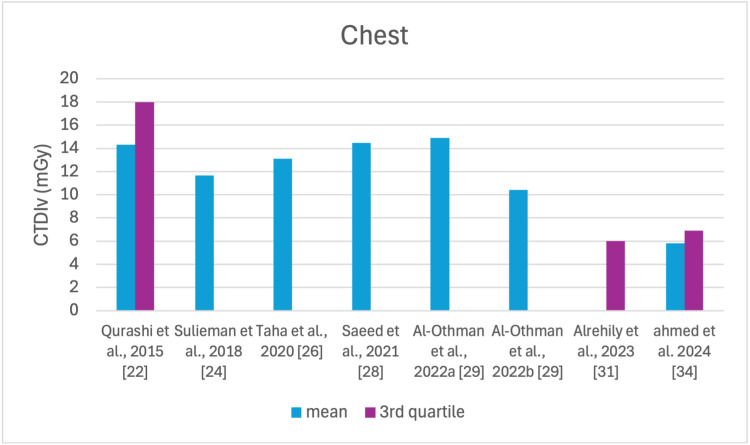
CTDIv reported by each study for the chest examination CTDI_v_: volumetric dose index

**Figure 4:**
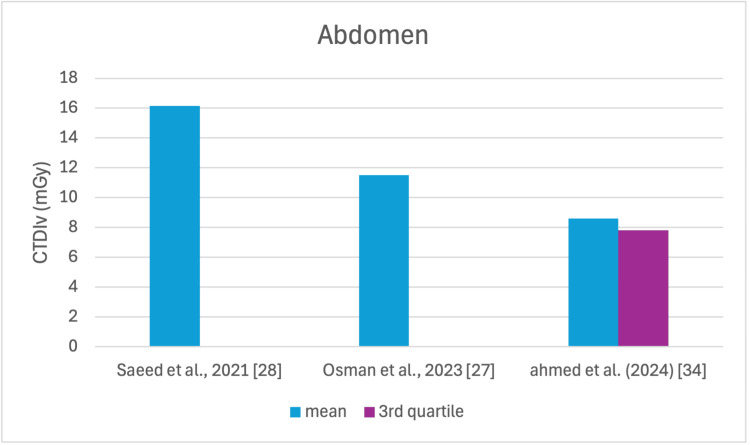
CTDIv reported by each study for the abdomen examination CTDI_v_: volumetric dose index

**Figure 5:**
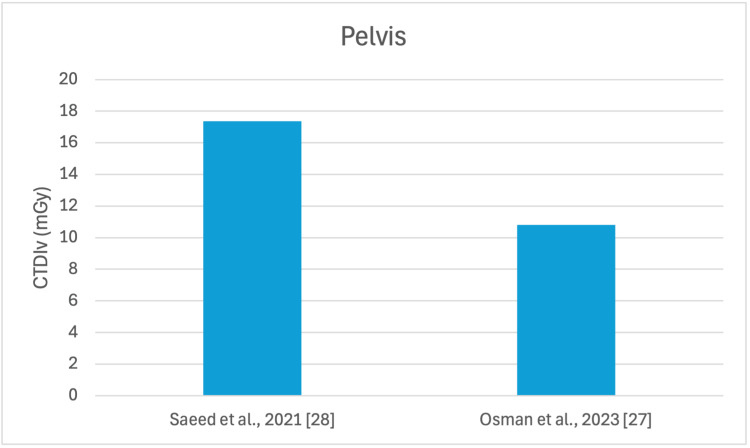
Mean CTDIv reported by each study for the pelvis examination CTDI_v_: volumetric dose index

**Figure 6:**
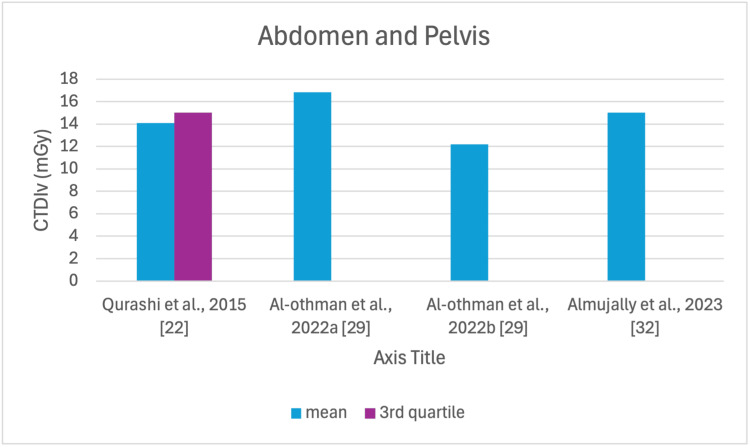
CTDIv reported by each study for the AP examination CTDI_v_: volumetric dose index, AP: abdomen and pelvis

**Figure 7:**
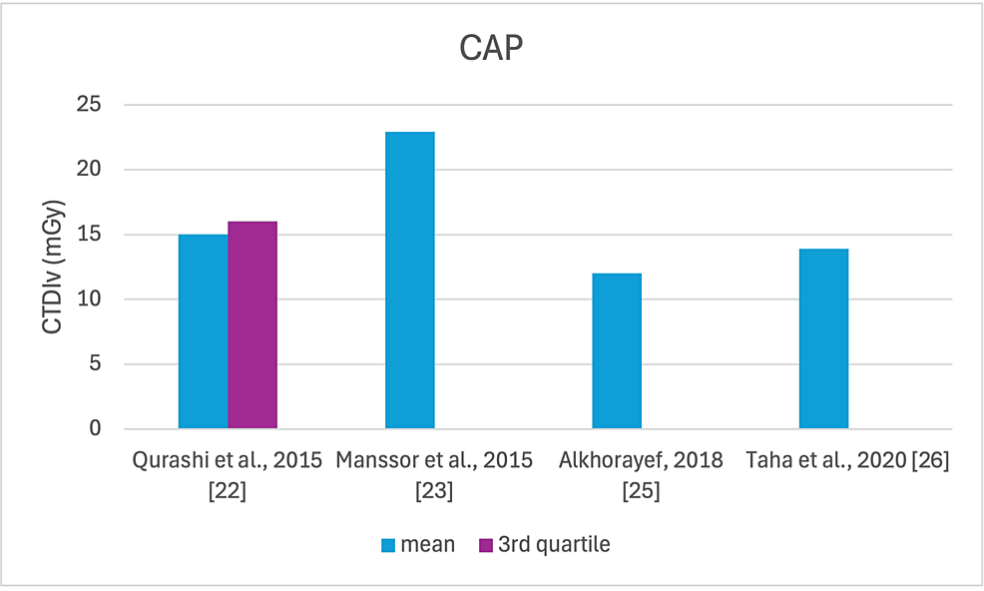
CTDIv reported by each study for the CAP examination CTDI_v_: volumetric dose index, CAP: chest, abdomen, and pelvis

**Figure 8:**
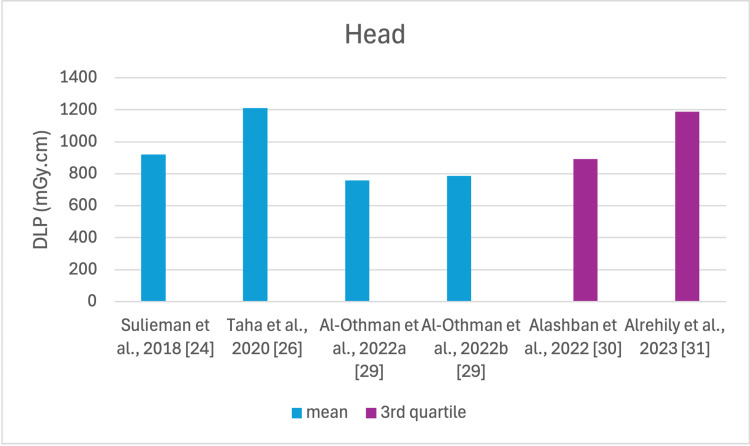
DLP reported by each study for the head examination DLP: dose length product

**Figure 9:**
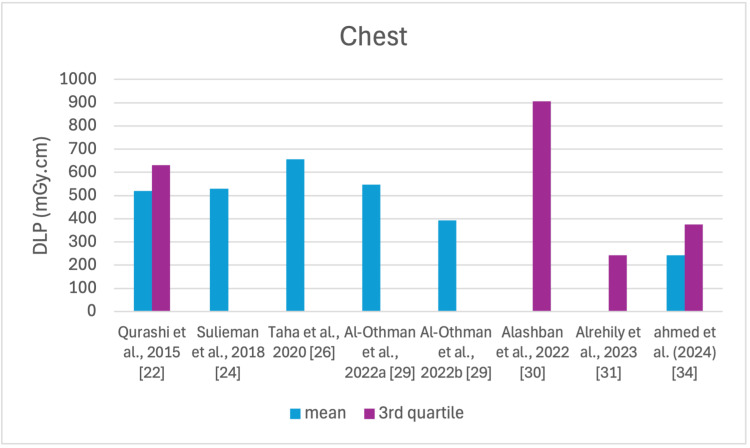
DLP reported by each study for the chest examination DLP: dose length product

**Figure 10:**
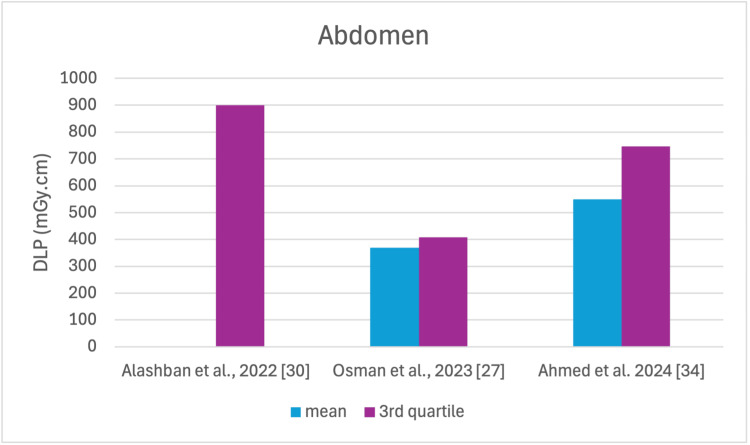
DLP reported by each study for the abdomen examination DLP: dose length product

**Figure 11:**
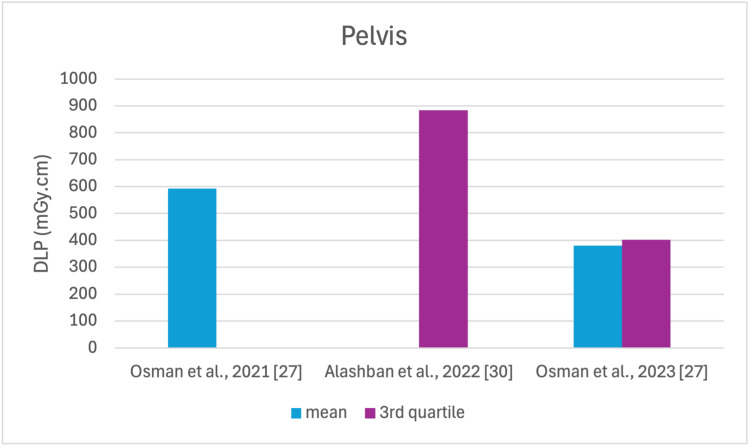
DLP reported by each study for the pelvis examination DLP: dose length product

**Figure 12:**
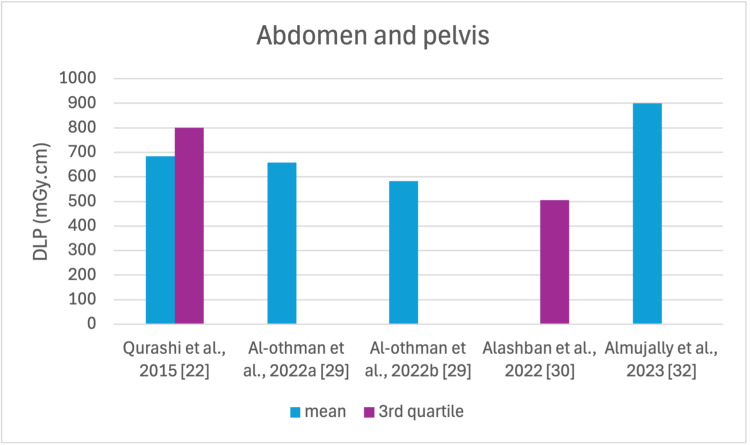
DLP reported by each study for the AP examination DLP: dose length product. AP: abdomen and pelvis

**Figure 13:**
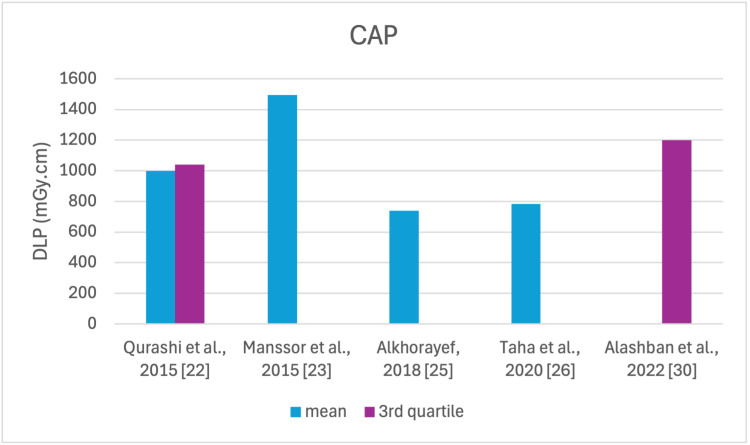
DLP reported by each study for the CAP examination DLP: dose length product, CAP: chest, abdomen, and pelvis

Discussion

In the last decade, CT scan utilization has increased significantly in medicine, resulting in an increased likelihood of radiation-induced cancers [35,36]. To address this concern, many strategies and methods have been initiated by manufacturers, hospitals, and health centers to reduce radiation doses. These strategies involve technological advancements like iterative reconstruction techniques, modulation of tube voltage and current, and the use of bow-tie filters [2].

To our knowledge, this is the first review that has been done to include radiation doses for adult CT examinations in Saudi Arabia. The review discovered that CTDI_v_ and DLP were the most frequently used as dosimetric quantities and the chest procedure was the most analyzed examination. The mean values in terms of CTDIv for the head, chest, abdomen, pelvis, AP, and CAP ranged from 40.67 to 61.80 mGy, 5.80 to 14.90 mGy, 8.60 to 16.15 mGy, 10.80 to 17.35 mGy, 14.10 to 16.84 mGy, and 12.00 to 22.94 mGy, respectively. The mean values in terms of DLP for the head, chest, abdomen, pelvis, AP, and CAP ranged from 757 to 1212 mGy.cm, 243 to 657 mGy.cm, 369.5 to 549 mGy.cm, 379.6 to 593 mGy.cm, 658 to 940.43 mGy.cm, and 740 to 1493.8 mGy.cm, respectively. 

This systematic review found a variation in the CTDI_v_ and DLP for the same type of examination without any clear pattern. This is unsurprising, considering that the majority of the studies were conducted within a relatively short period of time after 2020. For head scans, the CTDI_v_ and DLP had the highest mean values among other procedures, and both the chest and abdomen scans were the lowest.

Although the DRLs were determined using the second quartile (50th percentile), third quartile (75th percentile), and fourth quartile (90th percentile) values, it was globally observed that the 75th percentile was predominantly used across various studies to establish the DRLs [2]. The NDRL set by the SFDA for the head is 55 mGy and 1026 mGy.cm, AP is 14 mGy and 706 mGy.cm, and the chest is 12 mGy and 430 mGy.cm for CTDI_v_ and DLP, respectively [37]. Figures 2-13 show a few studies used 75th percentile CTDI_v_ for chest and abdominopelvic scans, and only Qurashi et al.'s study exceeded the NDRL with 18 and 15 mGy.cm, respectively [22]. For the head, only Alashban et al.'s and Alrehily et al.'s studies used the 75th percentile for DLP, with the latest exceeding the NDRL [30,31,37].

The findings indicate the head examinations had the lowest average values of ED ranging from 1.74 to 2.55 mSv. However, trunk scans had the highest average values ranging from 11.7 to 21.2 mSv. The review reveals that an increase in DLP directly affects the ED received by the patients since the ED is determined as the product of DLP and conversion factor. Therefore, it is crucial to limit the scan length to the region medically in question to avoid overscanning. Then, unnecessary exposure can be minimized, reducing the potential risks associated with radiation.

The American Association of Physicists in Medicine (AAPM) recommends conducting an annual review of CT scan protocols [38]. Although DRLs provide benchmarks for optimizing radiation exposure used in medical imaging examinations, they should not be used as strict limits. The primary objective of dose optimization is to minimize the dose as much as possible while ensuring the images retain their diagnostic quality. In certain situations, it may be necessary to utilize higher doses to obtain the necessary information for an accurate diagnosis [10].

The majority of studies have used mean values for dosimetric quantities. However, the review recommends utilizing the third quartile instead. This will facilitate the comparison of the DRLs at the national level. The SFDA published the NDRLs for only three CT examinations, so it is recommended that the NDRLs be reported for other routine examinations, such as abdomen, pelvis, and CAP CT scans. The review also suggests the importance of establishing DRLs based on weight, equivalent diameter, or cross-sectional area as suggested by the International Commission on Radiological Protection (ICRP) Publication 135. 

## Conclusions

The review concludes that the CTDI_v_ and DLP were the most commonly used dosimetric quantities, with the average and third quartile values being the most determined for dose comparison. The review also indicates that there has been a significant increase in adult patient dose surveys. However, the review identified fluctuations in radiation dose in terms of the CTDI_v_ and DLP values among CT centers, primarily since the majority of surveys were conducted in a relatively short period of time. The head scans had the highest mean CTDI_v_ and DLP, and the chest and abdomen scans had the lowest. For the same procedure, there is a linear relationship between the DLP and ED. As the scan length increases, the DLP also increases. Most studies have used average values for dosimetric measurements. However, the review recommends utilizing the third quartile instead to establish DRLs, expanding the national DRLs to include more routine examinations, and considering patient-specific factors, such as weight and size when establishing DRLs. Ongoing monitoring and optimization of CT protocols are crucial to minimize the potential risks associated with medical radiation exposure. Furthermore, there is a need to provide proper education and training to improve radiographers’ skills in limiting the scan area to the region of interest.
